# Feasibility of a Short-Arm Centrifuge for Mouse Hypergravity Experiments

**DOI:** 10.1371/journal.pone.0133981

**Published:** 2015-07-29

**Authors:** Hironobu Morita, Koji Obata, Chikara Abe, Dai Shiba, Masaki Shirakawa, Takashi Kudo, Satoru Takahashi

**Affiliations:** 1 Department of Physiology, Gifu University Graduate School of Medicine, Gifu, Japan; 2 Mouse Epigenetics Project, ISS/Kibo experiment, Japan Aerospace Exploration Agency, Tsukuba, Japan; 3 JEM Utilization Center, Human Spaceflight Technology Directorate, Japan Aerospace Exploration Agency, Tsukuba, Japan; 4 Laboratory Animal Resource Center, University of Tsukuba, Tsukuba, Japan; 5 Department of Anatomy and Embryology, Faculty of Medicine, University of Tsukuba, Tsukuba, Japan; Charles P. Darby Children's Research Institute, 173 Ashley Avenue, Charleston, SC 29425, USA, UNITED STATES

## Abstract

To elucidate the pure impact of microgravity on small mammals despite uncontrolled factors that exist in the International Space Station, it is necessary to construct a 1 *g* environment in space. The Japan Aerospace Exploration Agency has developed a novel mouse habitat cage unit that can be installed in the Cell Biology Experiment Facility in the Kibo module of the International Space Station. The Cell Biology Experiment Facility has a short-arm centrifuge to produce artificial 1 *g* gravity in space for mouse experiments. However, the gravitational gradient formed inside the rearing cage is larger when the radius of gyration is shorter; this may have some impact on mice. Accordingly, biological responses to hypergravity induced by a short-arm centrifuge were examined and compared with those induced by a long-arm centrifuge. Hypergravity induced a significant Fos expression in the central nervous system, a suppression of body mass growth, an acute and transient reduction in food intake, and impaired vestibulomotor coordination. There was no difference in these responses between mice raised in a short-arm centrifuge and those in a long-arm centrifuge. These results demonstrate the feasibility of using a short-arm centrifuge for mouse experiments.

## Introduction

To examine the impact of microgravity on a living body, many animal experiments have been performed on board the International Space Station [1–6]. Animals were launched into space, raised in microgravity conditions in the International Space Station, and retrieved. Data obtained from these animals were compared with that obtained from control animals, which were raised in 1 *g* conditions on Earth. Although the results of these experiments can be informative, other factors in the space environment than microgravity could affect the results, such as lack of convection, microbial environment [7,8], cosmic radiation [9,10], stressful conditions induced by launch and landing, and maybe some other factors likely still not suspected.

To overcome this flaw, the Japan Aerospace Exploration Agency has developed a novel mouse habitat cage unit that accommodates a single mouse per cage. This cage can be installed in the Cell Biology Experiment Facility in the Kibo module of the International Space Station. The Cell Biology Experiment Facility has two compartments: an artificial gravity section with a 15-cm arm centrifuge, and a microgravity section without the centrifuge. The artificial gravity section rotates at 77 rpm to produce artificial 1 *g* gravity in space. Control animals will be individually raised in the mouse habitat cage unit attached to the end of the arm with centrifugation at 77 rpm. That is, they will be launched into space and will be retrieved like the microgravity animals, but will be raised under an artificial 1 *g* environment in the International Space Station. The impact of pure microgravity can then be examined in defiance of other factors using this centrifuge.

However, the gravitational gradient formed inside the cage is larger and the Coriolis force is also larger if a short-arm centrifuge and high turning angle velocity are used. This may have some additional impact on the living body. Although a short-arm centrifuge (tern table) has been used for experiments using plants and cultured cells [11,12], there have been no experiments using this equipment with small mammals. Accordingly, the aim of this study was to examine biological responses to a short- and a long-arm centrifuge, and to evaluate the feasibility of a short-arm centrifuge for mouse experiments. The Japan Aerospace Exploration Agency is planning to send 12 mice (8-week-old male C57BL/6J) to the International Space Station: six mice for microgravity exposure and six mice as 1 *g* controls. The mice will spend 4 weeks in space. Thus, biological responses to a short- and a long-arm centrifuge were examined for 4 weeks.

## Materials and Methods

Animals used in the present study were maintained in accordance with the “Guiding Principles for Care and Use of Animals in the Field of Physiological Science” set by the Physiological Society of Japan. The experiments were approved by the Animal Research Committees of Gifu University and Japan Aerospace Exploration Agency. Male C57BL/6J mice (n = 52) were used for the experiment.

The experimental groups established in this study are summarized in Table 1. Animals were divided into three groups: Vestibular lesion (VL, n = 15), Sham (sham VL, n = 21), and Int (intact tympanum, n = 16). Six- to 7-week-old mice were anesthetized with an isoflurane (Escain, Pfizer, Tokyo, Japan) inhalation via a face mask (2%), and the VL surgery was performed through an external auditory meatus approach. After removal of the tympanic membrane, malleus, incus, and stapes, labyrinthine fluid was aspirated. A #20 or #25 file (Mani, Utsunomiya, Japan) was inserted into the oval window and surrounding bone was resected, and then electrical cautery was applied through the file [13]. For the sham VL surgery, the tympanic membrane was removed, but the auditory ossicles were left intact. In the Intact mice, no anesthesia or surgery was applied, and thus the tympanum was left intact. Five to 7 days after the sham or VL surgery, the success of the VL was confirmed by observing the swimming behavior of the mice [14]. Mice were placed on a sieve basket, and then the sieve basket was gently placed in a small tub filled with warm water. Mice with complete lesions were unable to determine the direction in which they had to swim, and continued to turn around under the water. Once we observed this behavior, the sieve basket was raised from the water immediately. No mice drowned or died as a consequence of the swimming test.

**Table 1:** Experimental groups established in this study.

Experiments	Groups of animals (n)	Protocol/measurements
Fos expression	Sham-1g (3), Sham-2g-S (3), Sham-2g-L (3), VL-2g-S (3)	Fos expression in the brain after 90 min of 2 *g* load. Behavior analysis by video monitor.
1.4 *g* for 4 weeks	Int-1g (6), Int-1.4g-S (6), Int-1.4g-L (4)	1.4 *g* for 4 weeks. Body mass, food intake, rotarod test.
1.4 *g* for 2 weeks	Sham-1g (4), Sham-1.4g-S (4), Sham-1.4g-L (4), VL-1g (4), VL-1.4g-S(4), VL-1.4g-L (4)	1.4 *g* for 2 weeks. Body mass.

Penicillin G potassium (3000 U/kg, Meiji Seika Pharma, Tokyo, Japan) and buprenorphine (3 μg/kg, Lepetan, Otsuka, Tokyo, Japan) were administered to the VL and the Sham mice subcutaneously prior to returning animals to their home cages. After recovery for 3 days from the VL or sham treatment, the mice were moved to individual cages (width 10 × 10 cm and height 8 cm), and maintained there for the remainder of the experiment. During the recovery from sham or VL surgery and during centrifugation, food was placed on the cage floor instead of on a wire bar lid.

The 1.4 *g* (for 2 or 4 weeks) and 2 *g* (for 90 min) environments were induced by centrifugation of custom-made gondola-type rotating box with a 15 cm short-arm (Advanced Engineering Service, Tokyo, Japan) or a 1.5 m long-arm (Shimadzu, Kyoto, Japan). To obtain 1.4 *g*, 77 rpm (rise time = 58 s, fall time = 21 s) for the short-arm or 24.4 rpm for the long-arm was applied (S1 File). To obtain 2 *g*, 101 rpm for the short-arm (rise time = 76 s, fall time = 26 s) or 32 rpm for the long-arm was applied. The individual cages were set in the rotating box. All the mice had access to food and water *ad libitum*, and the room temperature was maintained at 24 ± 1°C with a 12:12 h light-dark cycle. During a daily 30-min break, the cages were cleaned and water and food were refreshed. Behaviors of mice in the short-arm centrifuge were video-monitored during centrifugation.

### Fos expression in response to 2 *g*

The 8-week-old Sham and VL mice (1 to 2 weeks after sham or VL surgery) were placed in the short-arm (Sham-2g-S, n = 3; VL-2g-S, n = 3) or the long-arm (Sham-2g-L, n = 3) centrifugation and maintained in a 2 *g* environment for 90 min, as described above. At the end of the 2 *g* load, mice were deeply anesthetized with an isoflurane (3%) inhalation and perfused transcardially with 20 mL of heparinized saline, followed by 40 mL of 4% paraformaldehyde in phosphate buffer. The brain was then removed and stored in 4% paraformaldehyde at 4°C. The brain of the Sham mice maintained in 1 *g* was also removed (Sham-1g, n = 3). All specimens were stored overnight in 20% sucrose in phosphate-buffered saline at 4°C and then frozen on dry ice. Serial sections (40 μm) were cut with a cryostat (Leica CM1850; Leica Instruments, Wetzlar, Germany). Fos-containing cells were identified with an anti-c-Fos antibody (c-Fos Ab-5; Merck, Darmstadt, Germany) and the 3,3′-diaminobenzidine reaction. Hypergravity-induced Fos expression was examined in the nucleus of the solitary tract (NTS), the medial vestibular nuclei (MVe), the paraventricular hypothalamic nucleus (PVN), and the supraoptic nucleus (SON). The numbers of Fos-positive cells were counted by a researcher who was not involved in this experiment using ImageJ 1.47v. Furthermore, behavior of the Sham-2g-S and VL-2g-S mice was video recorded during the 2 *g* load.

### 1.4 *g* for 4 weeks

The 7-week-old Int mice were transferred to an individual cage (width 10 × 10 cm and height 8 cm) from a group-housing cage. In the individual cage, food was placed on the cage floor instead of on a wire bar lid. One week elapsed for acclimation to the cage, and then the mice were placed in the short-arm (Int-1.4g-S, n = 4) or the long-arm (Int-1.4g-L, n = 6) centrifugation and maintained in the 1.4 *g* environment for 4 weeks. The Int-1g mice (n = 6) were maintained in a normal 1 *g* environment. During a daily 30-min break, the body mass of the mice and the food intake were measured. Vestibular-related coordinated movement was estimated using rotarod (47600, Bioresearch Center, Nagoya, Japan). The mice were placed on the rotary rod with their heads facing against the direction of rotation. Rotation speed increased from 2 to 40 rpm in 2 min, and the mice were required to move forward to remain on the rod. The duration for which the mice remained on the rotating rod was measured. The rotarod test was conducted on days 3 and 4 before the start of centrifugation, and 30–60 min after the termination of centrifugation. The average of the former two was used as the pre-1.4 *g* value. The rotarod test for the Int-1g was performed on the same day as the Int-1.4g-S and the Int-1.4g-L.

### 1.4 *g* for 2 weeks

Eight-week-old Sham and VL mice were placed in the short-arm (Sham-S, n = 4; VL-S, n = 4) or the long-arm (Sham-L, n = 4; VL-L, n = 4) centrifugation and maintained in a 1.4 *g* environment for 2 weeks. During a daily 30-min break, the body mass of the mice was measured. Control mice were raised in a 1 *g* environment (Sham-1g, n = 4; VL-1g, n = 4).

At the end of the rotarod test (1.4 *g* for the 4-week group) or the end of centrifugation (1.4 *g* for the 2-week group), mice were anesthetized by inhalation of isoflurane (2%). A venous blood sample (0.8 mL) was withdrawn from the inferior vena cava, and then the mice were decapitated to obtain brain samples. Blood and brain samples were kept in a deep freezer (−80°C) for other studies.

#### Statistical analysis

All the data are presented as the mean ± SEM. Baseline body mass data in Table 2 were analyzed using an unpaired t-test. A one-way analysis of variance (ANOVA) was used for the data analysis of Fos-labeled cells. A repeated-measures two-way ANOVA was used for the data analysis of daily measurements of body mass and food intake, and rotarod test. If the *F* ratio indicated statistical significance, the Scheffe’s multiple comparison test was used for among-group comparisons. A value of *P* < 0.05 was considered statistically significant for the post hoc test.

**Table 2:** Baseline body mass (g) immediately before the 2-week 1.4 *g* load.

	1g	1.4g-S	1.4g-L
Sham	23.0 ± 0.5	23.4 ± 0.9	23.1 ± 0.5
VL	17.9 ± 0.8*	18.4 ± 1.7*	18.1 ± 0.4*

*P < 0.05 vs. Sham.

## Results

### Estimated and measured g gradient in the cage

**Fig 1:**
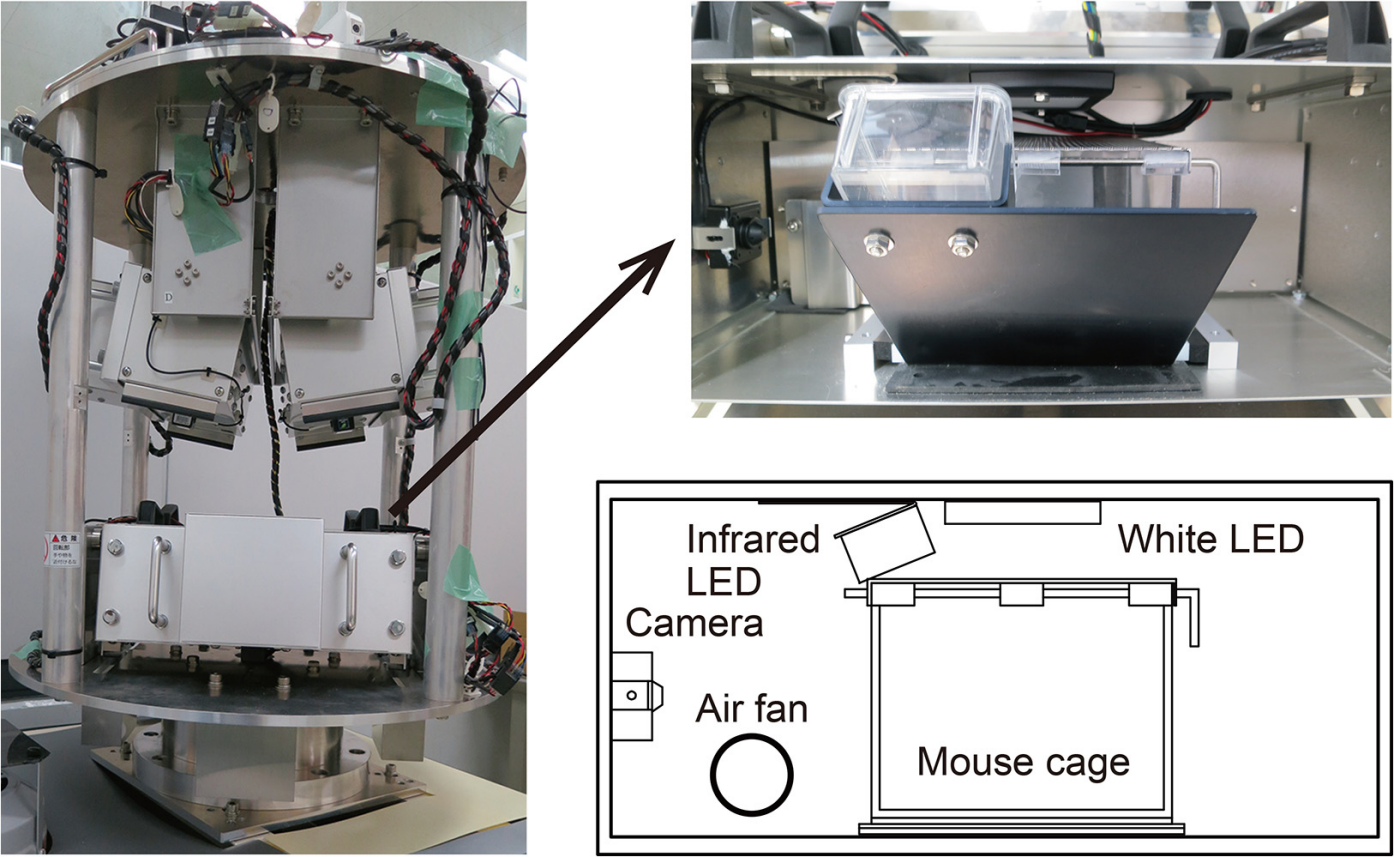
Photographs and a diagram of the newly-developed gondola-type short-arm centrifuge, which is equipped with 4 gondolas. Each gondola is equipped with a white LED, an infrared LED, a video camera for monitoring the behavior of mice, and an air fan for refreshing the air.

**Fig 2:**
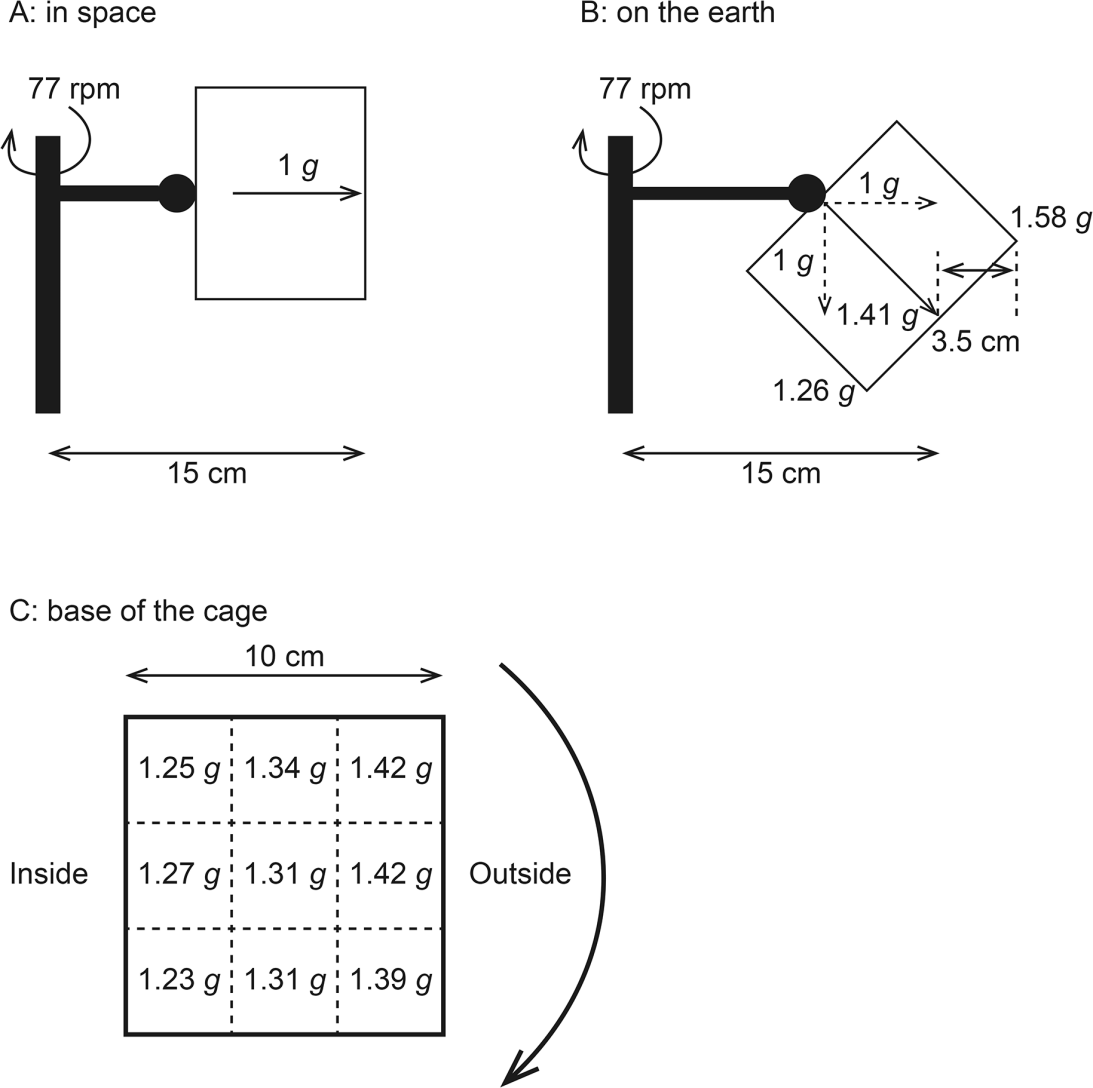
A: centrifugal force induced by centrifugation at 77 rpm in space. B: the synthetic gravity between 1 *g* terrestrial gravity and 1 *g* centrifugal force. C: the gravity gradient along the horizontal plane of the base of the cage.

The short-arm centrifuge is designed so that the radius of gyration at the center of the individual cage becomes 15 cm (Fig 1). Thus, centrifugation at 77 rpm produces a centrifugal force of 1 *g*, and this force acts in the dorsoventral direction of a mouse under the microgravity environment in space (Fig 2A). The radius of gyration shortens if the mouse rears up, and the centrifugal force at the head becomes small, because the centrifugal force is proportional to the radius of gyration. For example, if the head of a mouse is 5 cm above the base, the centrifugal force at the head becomes 0.66 *g*; this effect is negligible (0.97 *g*) if the long-arm (150 cm) centrifuge is used.

Under a 1 *g* environment on Earth, synthetic gravity (1.41 *g*) between 1 *g* terrestrial gravity and 1 *g* centrifugal force acts in the dorsoventral direction if a mouse is at the center of the cage (Fig 2B). The synthetic gravity changes if the mouse moves to the corner of the cage, because the radius of gyration changes. If the mouse moves to the outside corner, the radius of gyration becomes 18.5 cm and then the calculated synthetic gravity becomes 1.58 *g*. To examine this, the base of the cage was divided into nine areas, an accelerometer was set on the center of each area, and z-axis acceleration was measured. There was a gravity gradient of 0.19 *g* along the horizontal plane of the base (Fig 2C). The measured z-axis accelerations were less than the estimated synthetic gravity; this was probably because of the position of the acceleration sensor, which was approximately 2 cm above the base.

### Fos expression in response to 2 *g*

**Fig 3:**
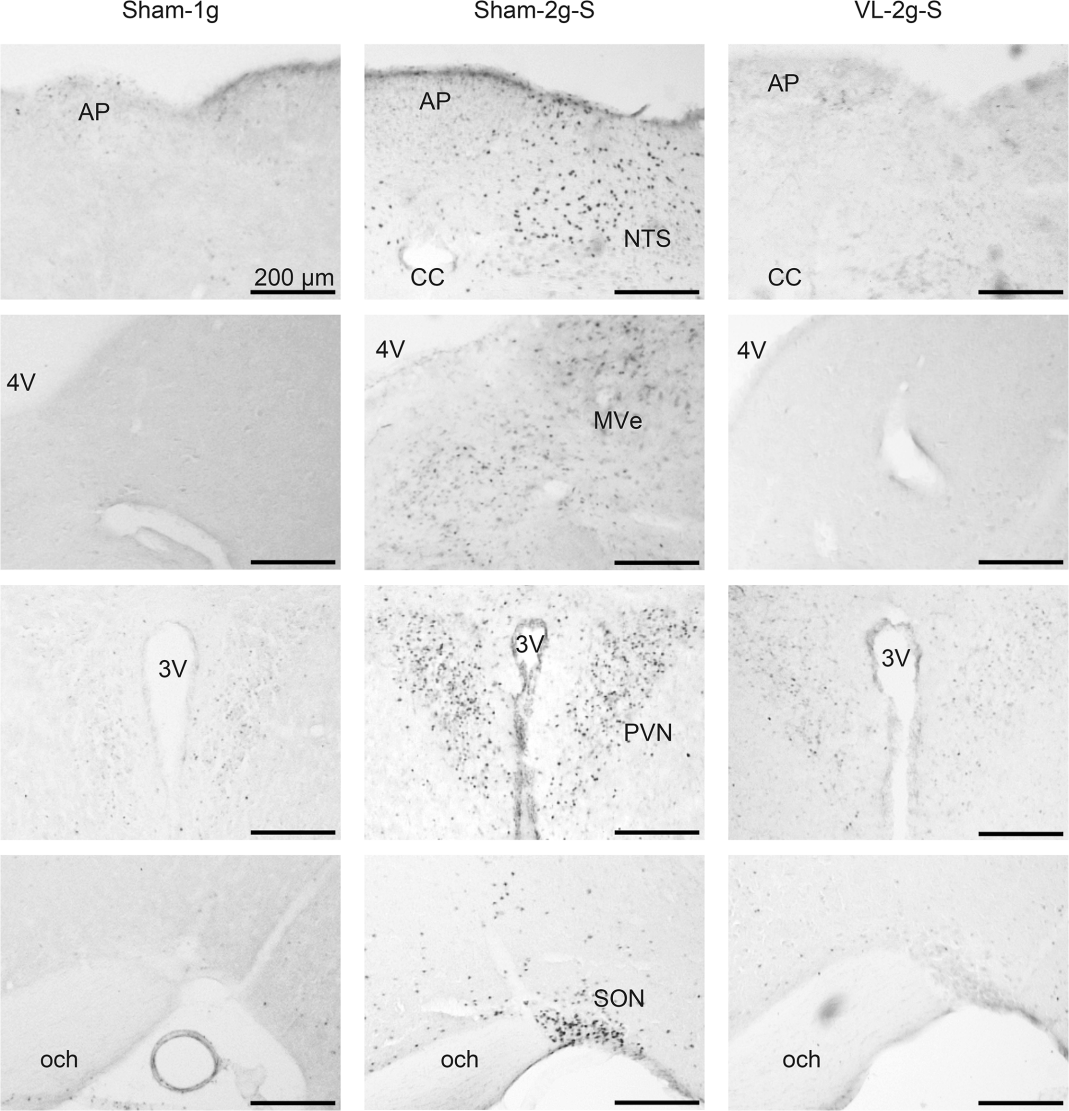
Typical photomicroscopic images of serial sections of the nucleus of the solitary tract (NTS), the medial vestibular nuclei (MVe), the paraventricular hypothalamic nucleus (PVN), and the supraoptic nucleus (SON) obtained from Sham-1g, Sham-2g-S, and VL-2g-S. AP: area postrema; 4V: 4th ventricle; 3V: 3rd ventricle; och: optic chiasm. −7.02 mm from the bregma for NTS, −5.88 mm from the bregma for MVe, −0.94 mm from bregma for PVN and SON.

**Fig 4:**
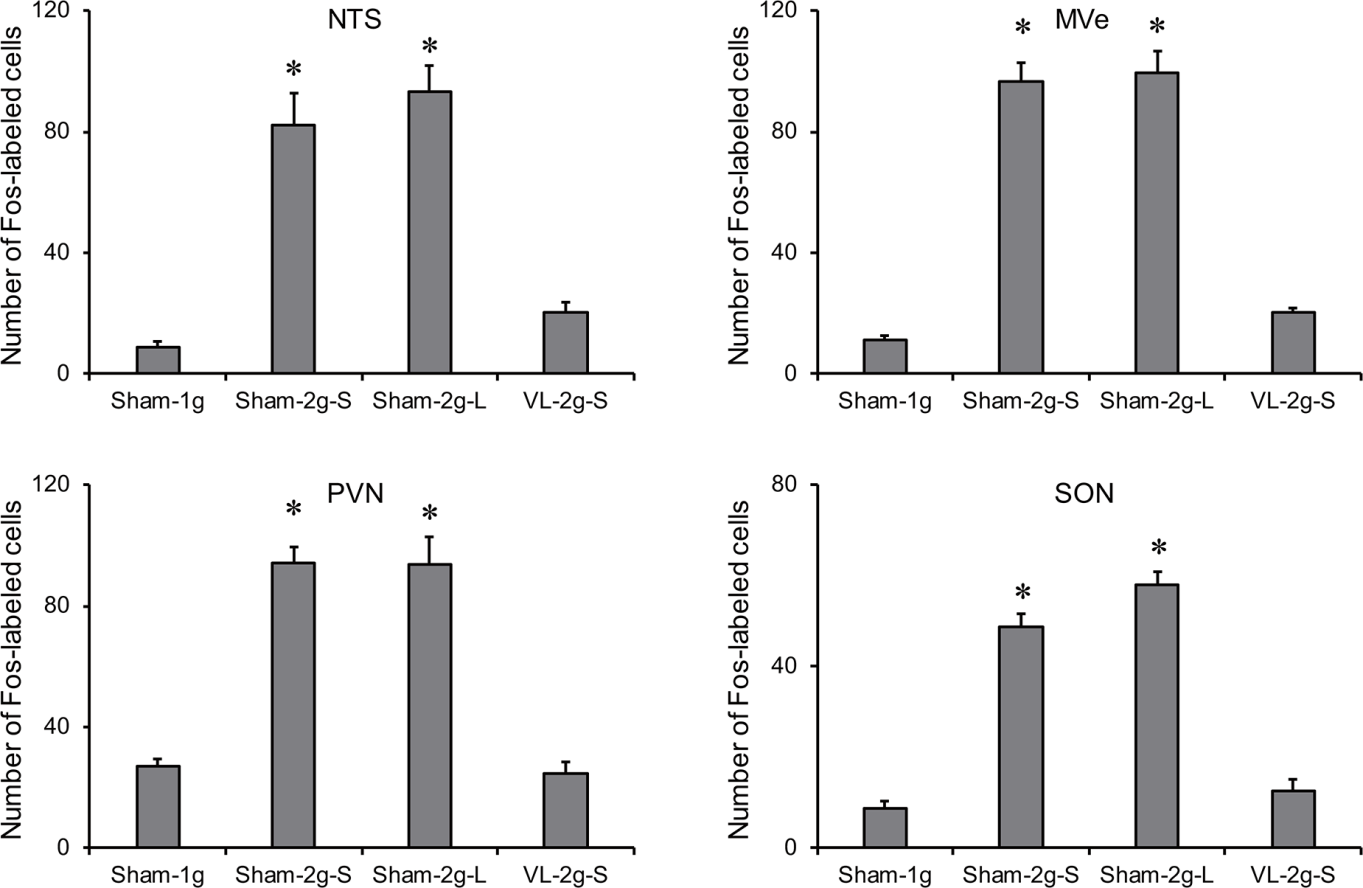
Summarized data for the numbers of Fos-labeled cells in the nucleus of the solitary tract (NTS), the medial vestibular nuclei (MVe), the paraventricular hypothalamic nucleus (PVN), and the supraoptic nucleus (SON) of Sham-1g, Sham-2g-S, Sham-2g-L, and VL-2g-S. In each nucleus, three to seven slices were picked up from each mouse, and then averaged within a group. The number of Fos-labeled cells in the NTS was the sum of the bilateral NTS, whereas the other nuclei were unilateral. *P < 0.05 vs. Sham-1g.

To examine central activation induced by the long-arm and the short-arm centrifugation, Fos expression in the NTS, MVe, PVN, and SON was compared among the Sham-1g, the Sham-2g-S, the Sham-2g-L, and the VL-2g-S groups. Fig 3 shows typical photomicroscopic images of serial sections obtained from the Sham-1g, the Sham-2g-S, and the VL-2g-S. Clear Fos expressions were evident in the Sham-2g-S, but only sporadically scattered Fos-labeled neurons were found in the Sham-1g or the VL-2g-S. Data for the numbers of Fos-labeled cells are summarized in Fig 4. In both the Sham-2g-S and the Sham-2g-L, the numbers of Fos-labeled cells in the NTS, MVe, PVN, and SON increased significantly compared with those in the Sham-1g, and no differences were found between the Sham-2g-S and the Sham-2g-L. These increases were completely abolished by VL, and no differences were found in the numbers of Fos-labeled cells in these areas between Sham-1g and VL-2g-S.

**Fig 5:**
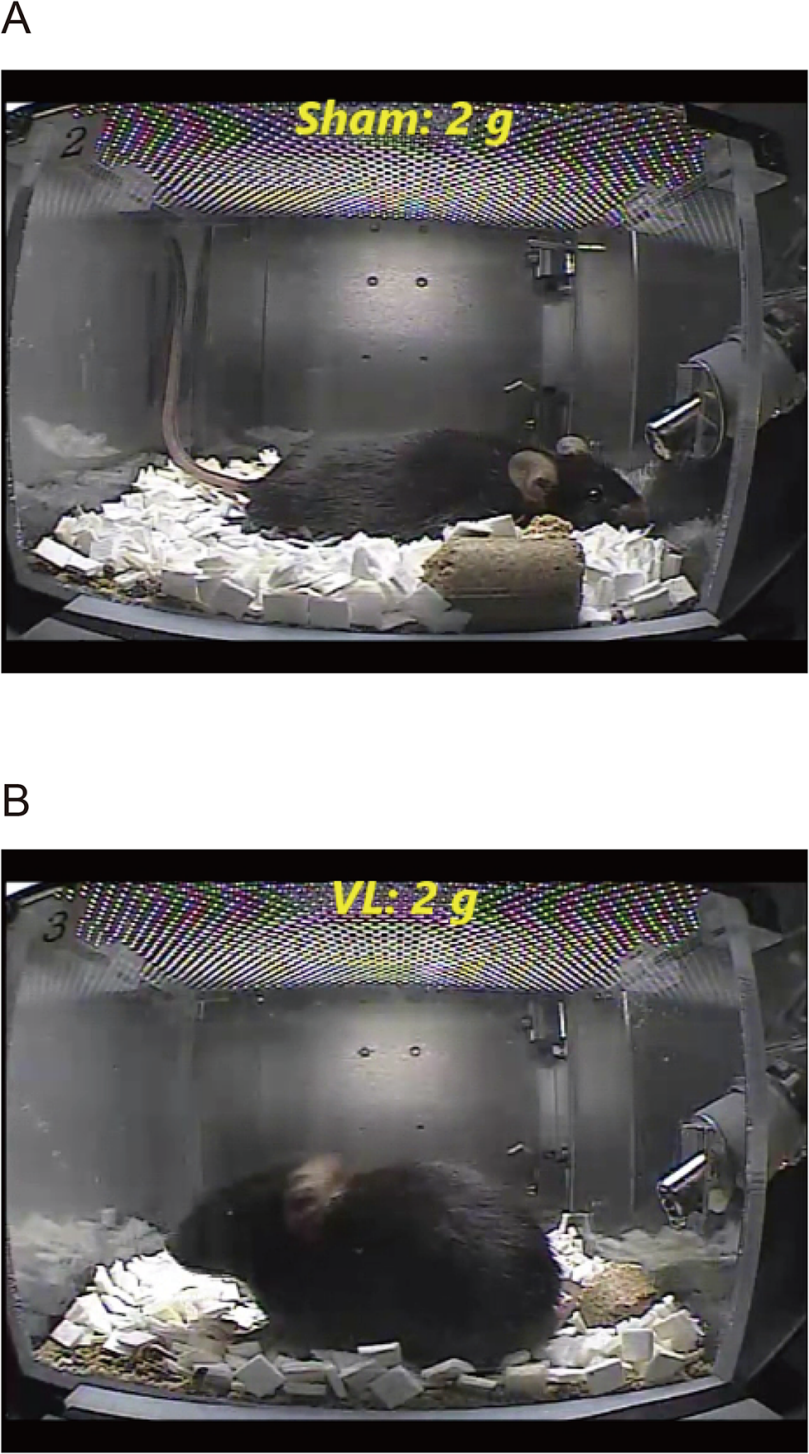
Posture at the onset of 2 *g* in a Sham (A) and a VL (B) mouse.

Behavioral analysis revealed that the round shape of the lumbar was lost, the trunk became flat, and the tail went up in all Sham-2g-S mice (Fig 5A and S2 File). This characteristic posture was not seen in the VL-2g-S mice, although they looked like they felt something and became somewhat restless at the onset of acceleration (Fig 5B and S3 File). Furthermore, all Sham-2g-S mice remained still and did not move during the 90-min 2 *g* period; however, VL-2g-S mice moved and sometimes reared up.

### 1.4 *g* for 4 weeks

**Fig 6:**
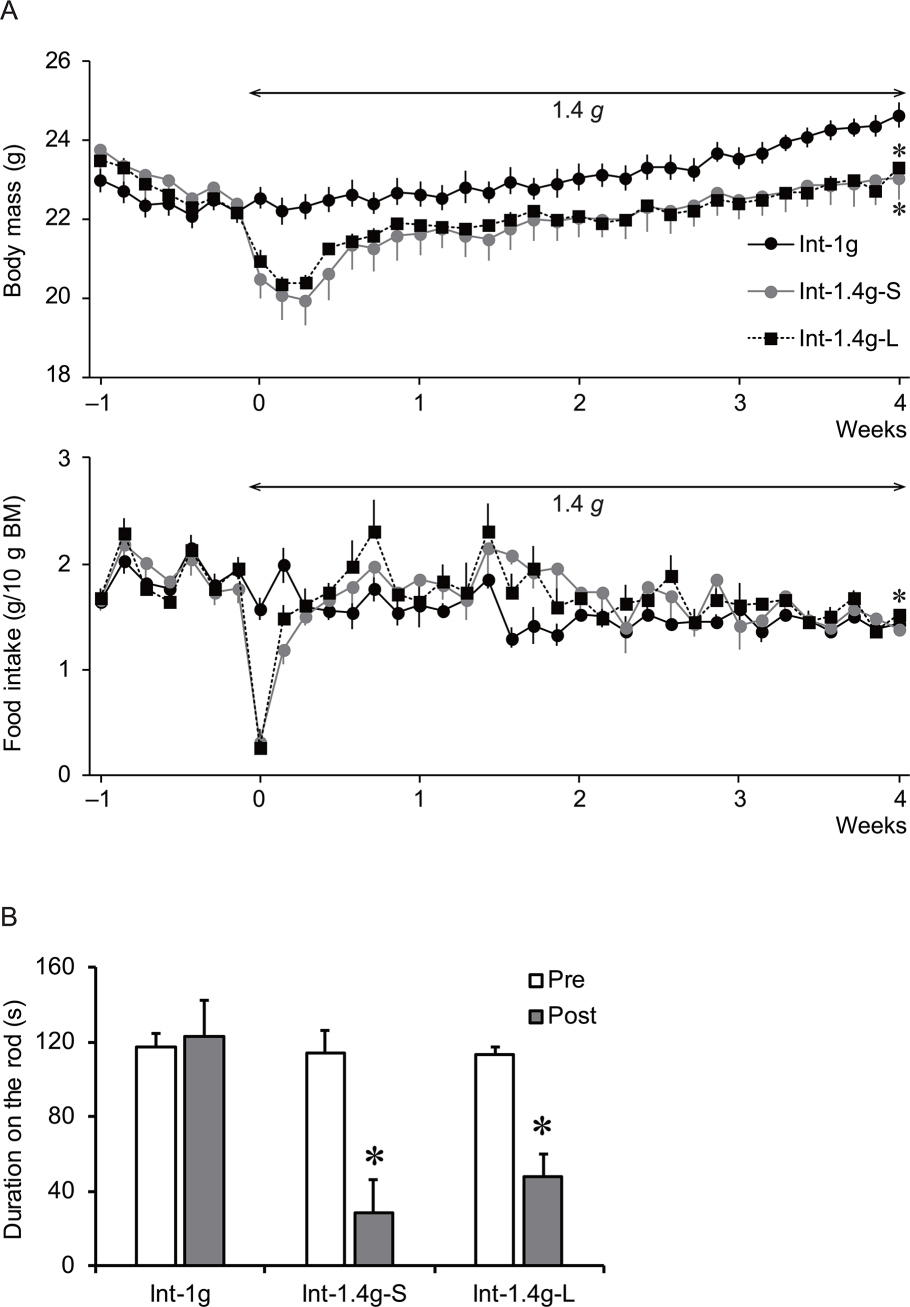
A: Summarized data for daily measurements of body mass and food intake per 10 g of body mass during the 1-week acclimation period and the subsequent 4-week 1.4 *g* period. * *P* < 0.05 vs. Int-1g mice. B: duration for which the mice could maintain themselves on the rotarod before and after the 4-week 1.4 *g* load. The open bar (Pre) and filled bar (Post) represent data obtained before and just after the 1.4 *g* load, respectively. * *P* < 0.05 vs. Pre.

No mice died during the 1.4 *g* experiments. Fig 6A shows daily measurements of body mass and food intake during the 1-week acclimation period and the subsequent 4-week 1.4 *g* period. In the Int-1g mice, body mass tended to decrease during acclimation to the individual cage, reached a plateau on day 5–7, and then gradually increased from 22.2 ± 0.3 to 24.6 ± 0.3 g at the end of the 4-week experimental period. In both the Int-1.4g-S and the Int-1.4g-L mice, their body masses decreased at the onset of 1.4 *g*, reaching a nadir (20.0 ± 0.7 g in Int-1.4g-S and 20.4 ± 0.2 g in Int-1.4g-L) on day 2 or 3, and then gradually increased. However, the body masses in the Int-1.4g-S (23.1 ± 0.5 g) and the Int-1.4g-L (23.3 ± 0.1) did not catch up with those in the Int-1g at the end of the experimental period. There was a significant interaction between group and time [*F*(2,70) = 10.573, *P* < 0.001]. The body mass responses of the Int-1.4g-S and the Int-1.4g-L were significantly different from those of the Int-1g, but no difference was found between the Int-1.4g-S and the Int-1.4g-L (*P* = 0.942).

During the acclimation period, the mice consumed 1.6–2.3 g/10 g body mass of food daily, and at the end of 4-week experimental period the Int-1g mice consumed 1.4 ± 0.1 g/10 g body mass. The 1.4 *g* load suppressed food intake for both the Int-1.4g-S (0.3 ± 0.1 g/10 g body mass) and the Int-1.4g-L (0.3 ± 0.2 g/10 g body mass) on day 1 of 1.4 *g*, but they soon recovered and caught up with that of the Int-1g on day 3 of 1.4 *g*. There was a significant interaction between group and time [*F*(2,70) = 3.066, *P* < 0.001]. The food intake of the Int-1.4g-L was significantly different from that of the Int-1g, but the difference between the Int-1.4g-S and the Int-1g did not reach statistical significance (*P* = 0.151). There was no significant difference in food intake between the Int-1.4g-S and the Int-1.4g-L (*P* = 0.943).

Fig 6B shows the duration for which mice could hold themselves on the rotating rod before and after the 4-week experimental period. The Int-1g mice remained on the rotating rod for 114 ± 8 s, which was not affected by the 4-week experimental period. However, the duration on the rod in the Int-1.4g-S and the Int-1.4g-L mice significantly decreased at the end of the experimental period. The durations on the rod of the Int-1.4g-S and the Int-1.4g-L were significantly different from that of the Int-1g, but no difference was found between the Int-1.4g-S and the Int-1.4g-L (*P* = 0.843).

### 1.4 *g* for 2 weeks

**Fig 7:**
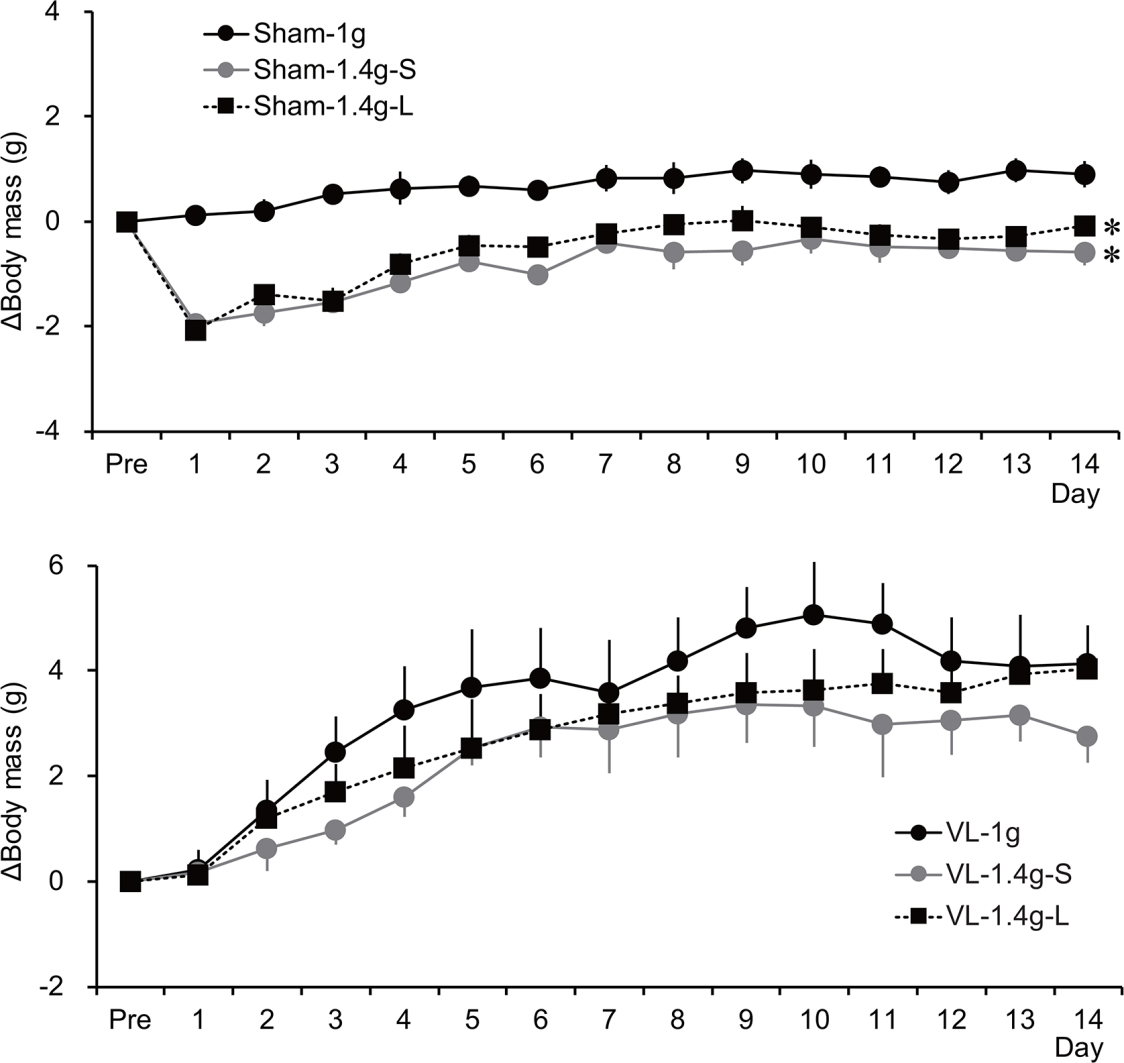
Summarized data for the daily changes in body mass during the 2-week 1.4 *g* load in the Sham (top) and the VL (bottom) mice. * *P* < 0.05 vs. Int-1g.

To examine the role of the vestibular system on hypergravity-induced reductions in body mass, this variable was measured for 2 weeks in the Sham and VL mice in a 1 *g* or 1.4 *g* environment (Fig 7). The body masses immediately before the experiment were significantly smaller in the VL mice than those in the Sham mice (Table 2). In the Sham mice, there was a significant interaction between group and time [*F*(2,28) = 6.094, *P* < 0.001]. The body masses of the Sham-1.4g-S and the Sham-1.4g-L were significantly different from those in the Sham-1g, but no difference was found between the Sham-1.4g-S and the Sham-1.4g-L (*P* = 0.529). Furthermore, no interaction between group and time was found in the VL mice [*F*(2,28) = 0.558, *P* = 0.471]. That is, the suppressive effect of hypergravity on body mass growth was abolished by VL.

## Discussion

The major findings of the present study were as follows. 1) The 1.4 *g* load suppressed food intake and reduced body mass growth, which was ameliorated by VL. 2) The 2 *g* load induced Fos expression in the NTS, MVe, PVN, and SON. This effect was also abolished by VL. 3) Although a larger gravitational gradient existed in the short-arm centrifuge compared with the long-arm centrifuge, there were no differences in food intake, body mass growth, the rotarod test, or Fos expression in response to hypergravity between the short-arm and the long-arm.

Previous hypergravity experiments from our laboratory using the long-arm centrifuge demonstrated that the body mass of the sham-treated rats was significantly decreased by the 3 *g* load [15]. This decrease was because of acute transient hypophagia, probably from hypergravity-induced motion sickness [13,15]. Hypophagia and the decrease in body mass were significantly ameliorated by VL, but a significant decrease was still observed compared with the rats raised in a 1 *g* environment. However in the present study, the decrease in body mass growth was completely abolished by VL. The difference between the present study and the previous studies might be based on differences in species (small body mass in mice) and the gravitational load (1.4 *g* vs. 3 *g*).

The vestibular system is known to be highly plastic; if it is exposed to a different gravitational environment, vestibular function is altered. Vestibular-mediated motor coordination is impaired in rats raised in microgravity or hypergravity environments [16,17]. To examine this, the rotarod test was performed before and after the 1.4 *g* load. The rotarod test, which evaluates the ability to maintain equilibrium on a rotating rod, has been used to examine vestibulomotor coordination [18]. The rotarod skill was impaired after the 1.4 *g* load both in the Int-1.4g-S and Int-1.4g-L mice, and no difference was found between the two groups of mice. Thus, impairments in vestibulomotor coordination were induced by both the short-arm and the long-arm centrifuge.

The 1.4 *g*-induced impairment of rotarod skill might be an adaptive response to 1.4 *g* rather than a deficit. Video behavior analysis revealed that the Sham-1.4g-S mice remained still at the onset of 1.4 *g*, the round shape of the lumbar was lost, the trunk became flat, and the tail went up. They started to move 1 to 2 hours after the start of centrifugation. Daily rear-up behavior of the Sham-1.4g-S was 24 ± 7 times/day at the 2nd day of 1.4 *g*, and it increased to 262 ± 31 times/day at the 7th day of 1.4 *g*. These results suggest that the vestibular system gradually adapted to the new gravitational environment, and that the Sham mice could learn to behave normally in a 1.4 *g* environment. If rotarod test was performed in a 1.4 *g* environment, the Sham mice might perform normally on the rod. This is a tremendously important and interesting issue that needs to be examined in a future study.

The 2 *g* load-induced Fos expression in the MVe was completely abolished by VL; this is reasonable, since the MVe receives projections from the peripheral vestibular organs [19]. However, it is unexpected that the hypergravity-induced Fos expression in the NTS, PVN, and SON were also completely abolished by VL, since hypergravity is sensed not only by the vestibular organs but also by the somatosensory, proprioceptive, and visceral sensors, which remained intact in the VL animals [20]. The hypergravity-induced increase in body weight (body mass × acceleration due to gravity) stimulates these sensors, and then might induce central Fos expression even in the VL animals. However, 2 *g* might not be large enough to stimulate nonvestibular sensors. In accordance with this, 2 *g* load-induced Fos expression in rats was completely abolished by VL, but significant Fos expressions were observed in the NTS and PVN in the 3 *g*-loaded VL rats, although the amount of Fos-labeled cells was significantly smaller compared with the 3 *g*-loaded intact rats [15,21].

To further examine this, the behavior of the Sham-2g-S and the VL-2g-S was examined. All Sham-2g-S mice showed the characteristic flat posture with their tails up. This posture might not be because of the increased body weight from the 2 *g* load, since this characteristic posture was not seen in the VL-2g-S mice. Thus, the 2 *g* load-induced flat posture was elicited by the vestibular sensation and the increased body weight per se had little impact on the behavior. Together with this and the Fos data, we suggest that nonvestibular sensation during the 2 *g* load might not be stressful for mice.

Although a larger gravity gradient existed in the short-arm centrifuge, biological responses to hypergravity—the suppression of the body mass growth, the decrease in food intake, the impaired vestibulomotor coordination, Fos expression in the central nervous system—were similar to those induced by the long-arm centrifuge. These descriptive results show the feasibility of using the short-arm centrifuge for mouse experiments. The impact of pure microgravity can be examined in defiance of other factors by using this centrifuge in the International Space Station. In other words, aside from the major effects of microgravity, effects of other uncontrolled factors that exist in the International Space Station can be evaluated by using the short-arm centrifuge.**Acknowledgments**

## Supporting Information

S1 File The long- and short-arm centrifuges, rotating 24.4 and 77 rpm to produce 1.4 *g*. (ZIP)

S2 File Sham mouse behavior during acceleration from 0 to 101 rpm to produce 2 *g*. (WMV ×8 speed play).
(ZIP)

S3 File VL mouse behavior during acceleration from 0 to 101 rpm to produce 2 *g*. (WMV ×8 speed play).
(ZIP)
